# Epigenetic Mechanisms of Plant Adaptation to Cadmium and Heavy Metal Stress

**DOI:** 10.3390/epigenomes9040043

**Published:** 2025-11-02

**Authors:** Eleonora Greco, Emanuela Talarico, Francesco Guarasci, Marina Camoli, Anna Maria Palermo, Alice Zambelli, Adriana Chiappetta, Fabrizio Araniti, Leonardo Bruno

**Affiliations:** 1 Department of Biology, Ecology and Earth Sciences (DiBEST), University of Calabria, 87036 Rende, Italy; eleonora.greco@unical.it (E.G.); emanuela.talarico@unical.it (E.T.); francesco.guarasci@unical.it (F.G.); marina.camoli@unical.it (M.C.); annamaria.palermo@unical.it (A.M.P.); adriana.chiappetta@unical.it (A.C.); 2 Department of Agricultural and Environmental Sciences-Production, Landscape, Agroenergy (Di.S.A.A.), University of Milano, 20133 Milano, Italy; alice.zambelli@unimi.it

**Keywords:** heavy metal stress, metalloid stress, cadmium (Cd) toxicity, plant stress response, epigenetics, transgenerational epigenetic memory, DNA methylation, histone remodeling, sustainable agriculture, epigenome editing

## Abstract

Heavy metal and metalloid stress, particularly from toxic elements like cadmium (Cd), poses a growing threat to plant ecosystems, crop productivity, and global food security. Elevated concentrations of these contaminants can trigger cytotoxic and genotoxic effects in plants, severely impairing growth, development, and reproduction. In recent years, epigenetic mechanisms have emerged as crucial regulators of plant responses to heavy metal stress, offering novel insights and strategies for enhancing plant resilience in contaminated environments. This review synthesises current advances in the field of plant epigenetics, focusing on key modifications such as DNA methylation, histone acetylation and remodelling, chromatin dynamics, and small RNA-mediated regulation. These processes not only influence gene expression under metal-induced stress but also hold promise for long-term adaptation through transgenerational epigenetic memory. Recent developments in high-throughput sequencing and functional genomics have accelerated the identification of epigenetic markers associated with stress tolerance, enabling the integration of these markers into breeding programs and targeted epigenome editing strategies. Special attention is given to cadmium stress responses, where specific epigenetic traits have been linked to enhanced tolerance. As plant epigenomic research progresses, its application in sustainable agriculture becomes increasingly evident offering environmentally friendly solutions to mitigate the impact of heavy metal pollution. This review provides a foundation for future research aimed at leveraging epigenetic tools to engineer crops capable of thriving under metal stress, thereby contributing to resilient agricultural systems and sustainable food production.

## 1. Introduction

The interaction between plants and their environment is complex and multifaceted, as plants are continuously exposed to various stresses. These stresses, which include both biotic and abiotic factors, compel plants to develop various adaptive strategies to survive. These strategies typically involve changes at the morphological, biochemical, and molecular levels [1,2]. One molecular aspect that has attracted significant attention is the alteration of epigenetic factors, which play a crucial role in plant responses to stress. Epigenetic changes can be induced by both biotic and abiotic stresses, influencing gene expression without altering the underlying DNA sequence [3,4]. Heavy metal and metalloid stress, including exposure to elements like cadmium (Cd), lead (Pb), mercury (Hg), and arsenic (As), represent significant threats to plant growth and development. These metals accumulate in soil and water, largely due to anthropogenic activities, and interfere with critical physiological and biochemical processes, leading to reduced growth, impaired photosynthesis, and overall poor plant health [5,6]. Plants respond to environmental stresses through multiple defense strategies, including activation of antioxidant systems and compartmentalization of toxic ions. Trans-cinnamic acid, a phenolic compound widely recognized for its role in plant metabolism, regulates diverse physiological and developmental processes [7].

Plants possess a range of protective mechanisms to cope with heavy metal stress, including activation of antioxidant systems, metal chelation, and compartmentalization. However, prolonged or excessive exposure can overwhelm these defenses, disrupting plant metabolism and reducing growth. Such effects manifest as decreased biomass, inhibited organ development, delayed germination or flowering, impaired photosynthetic efficiency, and nutrient imbalances [8]. Understanding these responses is essential for developing strategies to mitigate heavy metal toxicity and enhance plant resilience.

Cd, a toxic heavy metal, is a major environmental concern due to its detrimental impact on plant health, including soil degradation, reduced growth, and compromised physiological functions [9,10,11]. The accumulation of Cd in the environment is largely driven by human activities such as mining, the use of chemical pesticides, improper irrigation, and non-ferrous metallurgy [12,13]. Because of its non-biodegradable nature, high water solubility, and its ability to disrupt plant mineral uptake, Cd is considered one of the most hazardous soil contaminants [12,14]. In response to high Cd concentrations, plants have developed several protective mechanisms, such as limiting Cd uptake by roots, sequestering it in vacuoles, and synthesizing phytochelatins (PCs) that bind the metal [15,16,17]. These protective responses are regulated by specific genes, demonstrating the role of genetic programming in mediating stress tolerance.

In contrast to more commonly studied abiotic stresses such as drought and salinity, exposure to heavy metals and metalloids induces distinct plant responses. These responses include the synthesis of metal-binding molecules like PCs and metallothioneins (MTs), which sequester toxic metals and thereby reduce their toxicity rigorously [18]. Additionally, the enhancement of antioxidative defense mechanisms plays a critical role in mitigating oxidative stress caused by metal exposure. Key enzymes like superoxide dismutase (SOD), catalase (CAT), and various peroxidases help to scavenge reactive oxygen species (ROS) and maintain redox balance [19,20,21].

Transport proteins such as heavy metal ATPases (HMAs) and natural resistance-associated macrophage proteins (NRAMPs) are vital for the internal regulation of metal ions, facilitating their sequestration or efflux to prevent toxic accumulation level. These transport processes are closely coordinated with cellular signaling pathways, contributing to an integrated stress response that helps maintain cellular homeostasis [22].

Recent research in plant epigenetics has opened new avenues for enhancing plant resilience to environmental stressors, including metal toxicity. Epigenetic modifications, such as DNA methylation, histone modifications, and small RNA-mediated regulation, play a key role in regulating adaptive gene expression in response to changing environmental conditions [23,24]. These epigenetic changes enable plants to “remember” past stress encounters and mount more efficient responses to future stressors, a concept known as stress memory [25]. By modulating epigenetic pathways, scientists aim to improve plant tolerance to environmental challenges, including metal toxicity, which is an emerging area of interest for boosting crop resilience.

Phytoremediation, the process through which plants absorb, sequester, or detoxify contaminants, has shown promise as a strategy for addressing heavy metal pollution [26,27,28]. Modifying the plant epigenome offers a potential route for enhancing tolerance to environmental stressors, including heavy metals. This can be achieved through conventional breeding methods or advanced biotechnological tools like CRISPR (Clustered Regularly Interspaced Short Palindromic Repeats)-based epigenome editing, which allows for precise regulation of gene expression without altering the DNA sequence itself [29]. As our understanding of plant epigenetics deepens, the potential to develop resilient crop varieties that can thrive in challenging environments becomes increasingly feasible, offering solutions to global agricultural and environmental challenges [30].

In summary, the relationship between plants and their environment is highly intricate, and understanding the molecular mechanisms underlying plant responses to environmental stresses, particularly metal toxicity, is critical. Epigenetic mechanisms represent a promising area for enhancing plant resilience, and research in this field could lead to the development of crops capable of withstanding heavy metal contamination, contributing to more sustainable agricultural practices and ecological preservation [31,32].

The existing body of research emphasizes the critical role of epigenetic modifications in the regulation of gene expression, contributing significantly to how plants respond to environmental challenges. However, relatively few studies have explored the relationship between Cd exposure and epigenetic changes in plants [33]. This review, therefore, aims to delve into the various epigenetic mechanisms that govern crop plants’ responses to Cd toxicity.

## 2. Understanding Heavy Metal and Metalloid Stress

Metals and metalloids are elements that can become toxic when present in high concentrations. Metals (e.g., Pb, zinc (Zn), Cd) are generally good conductors of heat and electricity and widely used in industry, while metalloids (e.g., As) have properties that fall between metals and non-metals. Industrial processes such as smelting and refining are major sources of these pollutants, releasing them into air, water, and soil. Unlike organic contaminants, they do not degrade over time and instead accumulate in the environment.

Heavy metal pollution poses a serious threat to plant growth and agricultural productivity, and it presents significant risks to human health through bioaccumulation in the food chain, where toxic elements pass from soil and water into crops, animals, and ultimately humans [34]. Elevated concentrations of these substances in the environment can lead to various forms of phytotoxicity impacting plant growth, development, and reproduction, with symptoms such as stunted growth, wilting, leaf chlorosis, and necrosis eventually leading to decreased biomass and poor agricultural productivity [35,36,37]. In fact, these compounds, like other phytotoxic natural molecules, can impair photosynthesis, disturb nutrient uptake, and increase oxidative stress, collectively reducing plant growth and vitality [38]. For instance, excess Pb and Cd in soil can significantly impact chlorophyll synthesis, leading to leaf chlorosis and impaired photosynthetic capabilities, ultimately affecting growth and development [36,39].

It was reported that persistent exposure to high levels of heavy metals can exacerbate phytotoxicity, leading to genomic instability and programmed cell death (PCD) in extreme stress conditions [36,40,41]. Moreover, certain metals, such as Cd, can disrupt the balance of essential microelements in plants, adversely affecting enzyme activity and overall metabolic function [42,43].

To mitigate the effects of metal and metalloid toxicity, plants have evolved a variety of complex defense mechanisms. These include the activation of antioxidant systems, compartmentalization of toxic elements into vacuoles or other organelles, and the regulation of specific metal transporter proteins that control uptake and translocation of harmful ions [44,45,46]. Gaining insight into these biological pathways is essential for developing strategies to enhance plant tolerance to heavy metal stress, such as breeding or engineering crops with improved antioxidant capacity, metal sequestration, and stress-responsive gene regulation.

In recent years, advancements in omics technologies including genomics, transcriptomics, and proteomics have significantly enhanced our understanding of the genetic and molecular bases of plant responses to heavy metal and metalloid exposure [47]. In depth studies utilising these new generation holistic techniques have unveiled the molecular bases for plant adaptation mechanisms, illustrating how plants fine-tune their metabolic pathways in response to varying concentrations of heavy metals [48,49].

One promising strategy to address soil contamination is phytoremediation, the use of plants to extract, accumulate, or detoxify heavy metals from polluted environments [27,50,51]. To contend with heavy metal stress, plants have evolved intricate detoxification mechanisms. Some species, defined as hyperaccumulators, possess unique abilities to uptake, transport, and sequester heavy metals, thereby mitigating their toxic effects [48,52,53]. They employ strategies such as chelation and compartmentalization, often through specialized proteins like PCs and MTs, which bind heavy metals and facilitate their storage in vacuoles [53,54,55,56]. These adaptive traits are crucial not only for survival but also for enabling phytoremediation processes that utilize plants natural capabilities to extract, stabilize, or degrade pollutants from contaminated soils [57,58]. Moreover, studies have highlighted the importance of microbial interactions in enhancing plant tolerance to heavy metal stress. Endophytic bacteria and mycorrhizal fungi can promote plant health and improve heavy metal tolerance through various mechanisms, including increased nutrient uptake and enhanced antioxidant defense [59].

Despite these advances, several challenges remain in implementing effective and large-scale solutions for mitigating heavy metal and metalloid stress. Notably, there is a need for further research into the long-term effects of heavy metal exposure, the interaction between different environmental stressors, and the broader ecological consequences of remediation strategies. Addressing these gaps will be crucial for developing sustainable, scalable approaches to enhance plant resilience and protect ecosystems.

## 3. Sources and Types of Heavy Metals and Metalloids in the Environment

Heavy metals and metalloids are pervasive environmental pollutants that threaten ecosystems, agricultural productivity, and human health. Industrial activities, particularly those involved in metal processing, are among the primary contributors to environmental contamination. Facilities such as smelters and refineries emit substantial quantities of toxic metals, including Pb, Zn, and Cd, into the atmosphere and surrounding water systems [60,61]. Similarly, mining operations are major sources of environmental contamination, discharging heavy metals such as Hg, As, and nickel (Ni) into nearby soils and waterways. These pollutants can persist in the environment, leading to long-term degradation of terrestrial and aquatic ecosystems [62,63].

Agricultural practices also contribute to heavy metal accumulation in the environment. The prolonged use of chemical fertilizers and pesticides can introduce elements like copper (Cu) and As into the soil, which may accumulate over time. Additionally, agricultural runoff, often containing heavy metals from animal manure and sewage sludge, can contaminate water bodies and further exacerbate soil pollution [62,64,65].

Improper disposal of electronic waste and household products containing heavy metals represent another major source of contamination. These materials, when not managed properly, contribute to both soil and air pollution. Furthermore, industrial and municipal wastewater discharges introduce heavy metals into freshwater systems, negatively affecting aquatic biodiversity and water quality [60].

**Figure 1:**
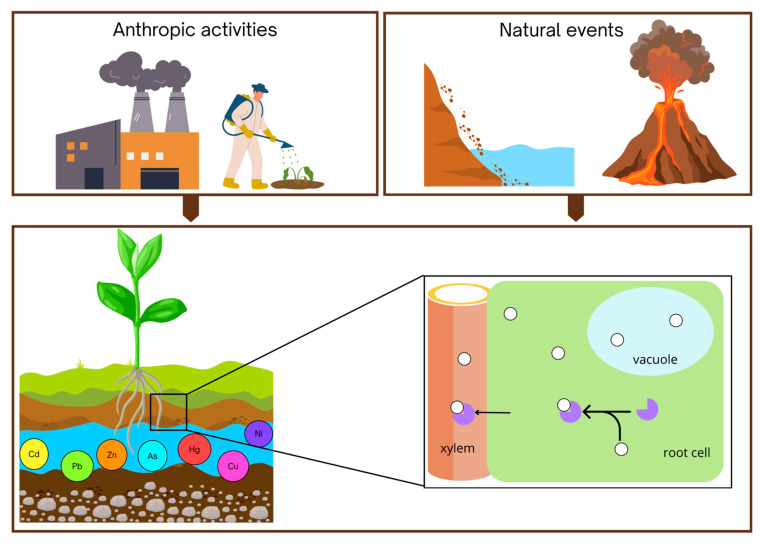
Major natural sources of heavy metal and metalloid contamination include rock weathering and volcanic activity, while anthropogenic sources primarily stem from industrial emissions and the extensive use of pesticides. Common contaminants such as cadmium (Cd), lead (Pb), mercury (Hg), arsenic (As), nickel (Ni), copper (Cu), and zinc (Zn) can persist in the environment and pose significant ecological risks. Once present in the soil, metal ions (indicated in white in the close-up) can be absorbed by plant roots, translocated to aerial tissues, and detoxified through mechanisms such as phytochelatin-mediated chelation, vacuolar sequestration, and activation of antioxidant defense systems. These physiological responses are fundamental to plant tolerance and form the basis for their application in phytoremediation strategies.

In addition to anthropogenic sources, certain natural processes also release heavy metals and metalloids into the environment. These include rock weathering, volcanic eruptions, and the breakdown of minerals, all of which can elevate concentrations of toxic elements in soil and water systems (Figure 1).

Heavy metals exist in various chemical forms and states within the environment, including soil, water, and atmospheric compartments. Their bioavailability and toxicity depend on numerous factors such as pH, redox potential, and interactions with organic and inorganic compounds. Understanding these dynamics is essential for accurately assessing environmental risk and implementing effective remediation strategies.

Heavy metals and metalloids present in the environment such as Cd, Hg, As, Pb, Cu, and Zn originate from a variety of interrelated sources (Table 1).

The distribution and interaction of these heavy metals and metalloids within the environment are complex and interconnected. A comprehensive understanding of their sources, mobility, and accumulation pathways is essential for devising effective environmental management strategies. This knowledge underpins the development of regulatory frameworks, pollution control measures, and sustainable practices aimed at mitigating their adverse effects on ecological systems, human health, and global environmental stability [34].

## 4. Impact of Heavy Metal and Metalloid Stress on Plant Growth and Development

Heavy metal and metalloid stress pose significant threats to plant growth and development, presenting major challenges to global agriculture and ecological sustainability. These toxic elements disrupt a range of physiological, morphological, and molecular processes, undermining plant vitality and productivity.

One of the most critical physiological impacts of heavy metal exposure is the inhibition of photosynthesis. Metals such as Cd and Pb interfere with chlorophyll biosynthesis and degradation, leading to decreased chlorophyll content and reduced light-harvesting capacity. Specifically, Cd and Pb toxicity can cause an imbalance by reducing the production of light-harvesting proteins like Lhcb, limiting light capture and impairing photosynthetic efficiency. These disruptions severely compromise plant metabolism and growth [71].

In parallel, metal stress induces the overproduction of ROS, resulting in oxidative stress. The accumulation of ROS leads to extensive cellular damage, including lipid peroxidation, protein denaturation, and DNA fragmentation, ultimately disrupting membrane integrity and enzyme functionality [71].

Heavy metals also affect nutrient dynamics by disrupting the uptake and assimilation of essential macro and micronutrients such as calcium (Ca), magnesium (Mg), and iron (Fe). This nutritional imbalance further hinders plant metabolism, enzyme activity, and cellular signalling [72].

Aboveground, symptoms such as chlorosis, necrosis, and leaf deformation reflect underlying cellular disruptions, including oxidative damage from ROS, membrane lipid peroxidation, and altered chloroplast ultrastructure [73,74,75,76,77].

Additionally, heavy metal contamination disrupts root soil microbe interactions essential for nutrient cycling and soil health, further compounding plant stress.

Reproductive development is also highly sensitive to heavy metal stress. Toxic concentrations reduce seed viability and germination rates, inhibit seedling establishment, and impair floral development, thereby compromising pollination and seed production [78,79].

At the molecular level, plants respond to metal stress through changes in gene expression and regulatory networks. Stress-responsive genes are activated, and plants may undergo epigenetic modifications, such as DNA methylation and histone modifications, that alter gene expression and may even transfer stress memory across generations [80,81,82]. Small RNAs (sRNA), particularly microRNAs (miRNAs), also contribute by regulating gene expression involved in stress adaptation [83].

To counteract oxidative stress, plants activate both enzymatic and non-enzymatic antioxidant systems. Key antioxidant enzymes include SOD, CAT, and peroxidase, which detoxify ROS. Non-enzymatic antioxidants like glutathione and ascorbate serve as vital scavengers, adding another layer of defense [84,85].

Another important defense mechanism is metal detoxification via vacuolar sequestration. Plants compartmentalize heavy metals in vacuoles, reducing their interaction with sensitive cellular components [86,87]. Chelating molecules such as PCs and MTs bind to metal ions, forming fewer toxic complexes that can be transported or stored safely [88].

Furthermore, plants engage sophisticated signaling pathways, including mitogen-activated protein kinase (MAPK) cascades, which orchestrate cellular responses to environmental cues and regulate stress-adaptive behaviors [89,90]. The activation or repression of metal responsive genes enables plants to reprogram metabolic processes and enhance their stress tolerance.

In some cases, certain plant species exhibit natural adaptations such as hyperaccumulation, allowing them to thrive in metal-contaminated soils by efficiently absorbing and compartmentalizing heavy metals [91]. These hyperaccumulators are of interest for phytoremediation efforts.

Understanding the multifaceted impact of heavy metal and metalloid stress from physiological disruption to molecular adaptation is essential for developing effective mitigation strategies. Future research should focus on exploiting these insights to engineer or breed crops with enhanced tolerance, promoting sustainable agriculture and addressing the environmental consequences of metal pollution.

## 5. Epigenetic Mechanisms in Plant Resilience to Heavy Metal Stress

**Figure 2:**
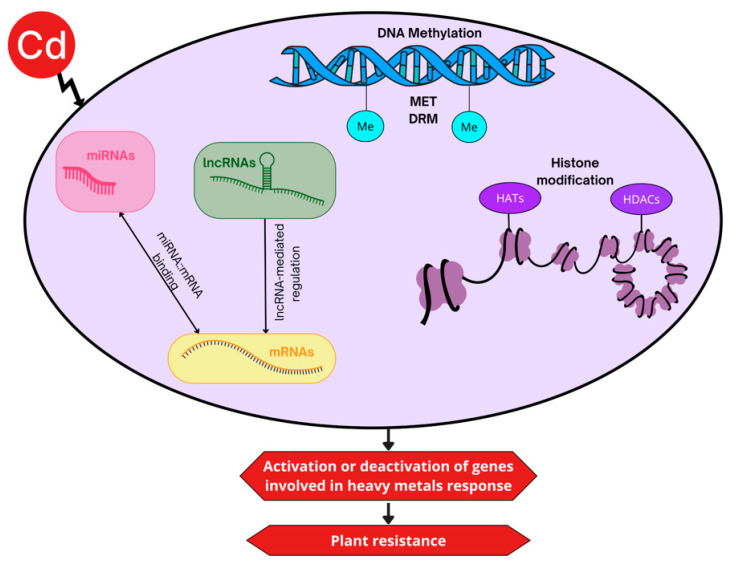
Schematic representation of key epigenetic mechanisms involved in the plant response to cadmium (Cd) stress. DNA methylation, histone modifications, and microRNAs (miRNAs) dynamically and reversibly regulate gene expression in response to Cd exposure. These mechanisms support rapid acclimation to stress conditions and may also contribute to transgenerational stress memory, enhancing long-term plant resilience.

Plants have evolved sophisticated epigenetic mechanisms to adapt to environmental stresses, including heavy metal and metalloid toxicity, without altering their underlying DNA sequence [32]. These mechanisms regulate gene expression through chemical modifications of DNA, histones, and associated regulatory RNAs, allowing plants to rapidly respond to adverse conditions and, in some cases, transmit adaptive information to subsequent generations (Figure 2).

### 5.1. DNA Methylation

DNA methylation, primarily occurring at cytosine residues within CpG dinucleotides, is catalyzed by DNA methyltransferases (DNMTs) [24,92,93,94,95]. This modification can either activate or repress gene expression depending on its genomic context, particularly when present in promoter regions of stress-responsive genes [96,97]. Demethylation can induce expression of protective genes, whereas hypermethylation can silence genes whose activity might exacerbate stress-induced damage. Notably, certain methylation patterns are faithfully maintained through mitotic divisions by the action of maintenance DNA methyltransferases such as METHYLTRANSFERASE 1 (MET1), which recognize hemi-methylated DNA strands after replication and restore full methylation. This stability ensures that stress-induced epigenetic marks persist across cell generations. Moreover, in some cases, these marks escape epigenetic reprogramming during gametogenesis and embryogenesis, allowing their transmission to progeny and providing a molecular basis for transgenerational stress memory [24,92,93,94,95].

### 5.2. Histone Modifications

Histone modifications regulate chromatin accessibility and transcription: acetylation by HATs promotes gene ex-pression, whereas deacetylation by HDACs represses it. Similarly, HISTONE MONOUBIQUITINATION1 (HUB1) and its paralog HUB2 form a conserved complex that modulates developmental programs, including flowering, dormancy, and the circadian clock, through H2B monoubiquitination (H2Bub) [98]. Histone methylation can exert either activating or repressive effects depending on the specific residue and methylation state. Among histone variants, H2A.Z is particularly important because it influences nucleosome stability and transcriptional responsiveness under normal growth conditions. Under stress, the incorporation of H2A.Z, along with dynamic histone replacement, fine-tunes chromatin accessibility and enables precise regulation of stress-responsive genes [99,100].

### 5.3. Non-Coding RNAs

Non-coding RNAs (ncRNAs) provide an additional layer of epigenetic regulation. MiRNAs modulate gene expression by directing degradation or translational inhibition of target mRNAs [101]. Small interfering RNAs (siRNAs) participate in RNA-directed DNA methylation (RdDM), reinforcing locus-specific epigenetic silencing [102]. Emerging evidence also implicates long non-coding RNAs (lncRNAs) in coordinating transcriptional networks that influence progeny traits and long-term stress adaptation [103].

### 5.4. Stress Memory and Transgenerational Epigenetic Inheritance

Stress-induced epigenetic modifications can persist after the initial exposure, allowing plants to “remember” previous stress events and mount faster and stronger responses upon re-exposure [97]. This phenomenon, known as stress memory, not only enhances immediate resilience but, in some cases, can be transmitted to offspring, forming transgenerational stress memory that improves progeny tolerance and contributes to population-level adaptation in metal-contaminated environments [103,104,105,106,107,108,109,110,111,112].

### 5.5. Epigenome Editing and Applications in Crop Improvement

Recent advances in CRISPR-dCas9-based epigenome editing allow precise modification of DNA methylation and histone marks without altering the underlying DNA sequence [113,114]. These tools offer promising strategies for engineering crops with enhanced heavy metal tolerance, improved yields, and greater resilience to environmental stresses. Integrating epigenetic insights into breeding programs presents an opportunity to develop sustainable, stress-resilient crop varieties capable of thriving under adverse environmental conditions [115,116].

## 6. Transgenerational Epigenetic Inheritance and Stress Memory

Plants can “remember” environmental stress across generations through epigenetic mechanisms that do not alter DNA sequences. Exposure to heavy metals such as Cd, Pb, As, and Hg can reprogram DNA methylation, histone modifications, and small RNA (sRNA) activity, resulting in heritable changes in gene expression that enhance progeny tolerance [104,105]. This phenomenon, often described as stress memory, provides adaptive advantages in contaminated environments where selective pressures persist.

In rice, Cd exposure has been shown to induce transgenerational changes in DNA methylation, improving tolerance in subsequent generations [104]. Similarly, altered expression of stress-responsive genes mediated by sRNAs contributes to better growth under Cd stress [107]. Recent studies highlight the role of lncRNAs in coordinating transcriptional networks that affect progeny traits, broadening the scope of RNA-mediated inheritance [103].

Epigenetic reprogramming of the germline enables the transmission of these altered regulatory states, influencing not only stress resilience but also development, phenology, and susceptibility to other environmental challenges [108,109,110,111]. At the population level, stress memory can enhance community-wide resilience, shaping ecological dynamics in metal-polluted soils [112]. Despite these insights, the integration of siRNAs, lncRNAs, and chromatin remodelers into heritable epigenetic memory remains poorly understood, and elucidating these mechanisms will be crucial for breeding programs aiming to improve crop tolerance without permanent genetic modification.

## 7. Chromatin Remodeling Under Cadmium Stress

Chromatin structure is a central regulator of gene expression, and its remodeling is a key adaptive response under Cd toxicity. Cd stress induces global chromatin alterations, including histone modifications, nucleosome repositioning, and changes in chromatin assembly pathways, which collectively reshape the transcriptional landscape.

Two major chromatin assembly systems, the histone chaperone HIRA and the CAF-1 complex, are disrupted under Cd stress, compromising replication fidelity and genome stability [117]. Cd can also bind thiol groups of histones and nuclear proteins, causing structural changes that impair DNA replication and transcription [118]. Studies in plants demonstrate that Cd exposure alters histone acetylation and methylation, affecting chromatin accessibility and activating stress-responsive pathways [119].

For instance, in *Arabidopsis*, CAF-1–mediated deposition of acetylated histones is critical for chromatin reassembly during Cd-induced replication stress [120]. In *Vicia faba*, Cd treatment triggers ROS production and S-phase chromatin reorganization, highlighting the link between oxidative stress and chromatin destabilization [121]. Collectively, these findings show that Cd compromises genome integrity while inducing compensatory chromatin remodeling to activate tolerance mechanisms. Understanding these structural effects is essential for advancing plant biotechnology and developing strategies to enhance Cd tolerance in crops.

## 8. The Role of DNA Methylation in Cadmium Stress Response

DNA methylation, involves adding a methyl group to the fifth carbon of cytosine residues, influencing gene expression and maintaining genome integrity [122]. This modification is reversible, allowing plants to dynamically regulate gene activity in response to developmental signals and environmental stresses, including heavy metal exposure [33] (Figure 2). DNA methylation is conserved across plant taxa, from mosses and ferns to flowering plants, highlighting its fundamental role in growth, development, and stress adaptation [123,124]. Methylation in promoter regions can modulate transcription of stress-responsive genes, thereby affecting physiological responses to adverse conditions [125].

Evidence from wheat (*Triticum aestivum*) demonstrates the functional relevance of DNA methylation under Cd stress. Shafiq et al. (2019) compared a Cd-tolerant wheat cultivar (Pirsabak 2004) with a Cd-sensitive one (Fakhar-e-Sarhad) and found that the sensitive cultivar exhibited elevated promoter methylation of metal transporter genes, including *ATB-binding Cassette subfamily C* (*TaABCCs*) and *Heavy Metal ATPase 2* (*TaHMA2*) [10,126]. In contrast, the tolerant cultivar showed lower methylation at these loci, suggesting that reduced methylation facilitates higher gene expression and more efficient Cd transport, contributing to improved metal tolerance.

Similarly, studies in *Arabidopsis thaliana* show that Cd exposure can increase overall DNA methylation, as observed in sensitive wheat cultivars [12]. In a triple mutant line lacking key DNA demethylases (ROS1, DML2, DML3), plants displayed enhanced growth under high Cd concentrations (>20 μM). This enhanced tolerance was associated with higher DNA methylation and improved Fe uptake, suggesting a feedback loop where methylation not only regulates stress-responsive genes but also modulates nutrient homeostasis under Cd stress.

These findings underscore the complex and context-dependent role of DNA methylation in plant responses to Cd. Methylation patterns can either repress or activate stress-responsive genes, influencing a plant’s capacity to tolerate metal toxicity. Differences between tolerant and sensitive cultivars indicate the potential of targeted epigenetic manipulation to improve crop resilience.

Further research is required to fully unravel how DNA methylation integrates with other epigenetic mechanisms, such as histone modifications and small RNAs, to mediate Cd tolerance. Understanding these pathways will be pivotal for developing crops capable of thriving in contaminated or stress-prone environments.

## 9. Impact of Cadmium Stress on Histone Acetylation

While global chromatin remodeling sets the stage, Cd stress also triggers gene-specific epigenetic regulation, particularly through histone acetylation. Histone acetylation, mediated by HATs (histone acetyltransferases) and removed by HDACs (histone deacetylases), regulates gene transcription by altering chromatin accessibility [127,128]. This reversible modification is central to processes such as growth, flowering, seed development, and responses to abiotic stresses including heavy metals.

In maize, combined exposures to Cd, Pb, and Zn influence both DNMTs and HDAC expression, modulating promoter methylation and histone acetylation at *ZIP* (Zn-regulated, iron-regulated transporter-like protein) transporter genes [119]. For example, several *ZIP* genes (*IRT1*, *ZIP1*, *ZIP3*, *ZIP5*, *ZIP6*, *ZIP7*) showed inverse correlations between HDAC expression and transcription levels, whereas *ZIP2* and *ZIP8* displayed positive correlations with both HDACs and DNMTs, suggesting alternative regulatory pathways. These findings highlight the intricate crosstalk between histone acetylation, DNA methylation, and metal transporter expression, which fine-tunes plant responses to toxic metals.

From a practical perspective, these results caution against the overuse of Zn fertilizers in Cd-contaminated soils, as such interactions may exacerbate metal uptake and disrupt nutrient homeostasis. Overall, understanding gene-specific histone acetylation provides insight into epigenetic mechanisms that can be leveraged to improve heavy metal tolerance in crops.

## 10. MicroRNAs and Their Role in Cadmium Tolerance

MicroRNAs (miRNAs) are short, non-coding RNA molecules that guide post-transcriptional gene silencing by binding to target mRNAs, leading to their degradation or translational inhibition [129]. These sRNAs act as crucial epigenetic regulators, modulating protein synthesis without altering the underlying DNA sequence [130] (Figure 2). MiRNA-mediated regulation is particularly important in plant responses to environmental stresses, including exposure to heavy metals like Cd [129]. Despite growing evidence on their involvement, the precise regulatory roles and mechanistic contributions of miRNAs in Cd detoxification are still being elucidated.

Previous research has established that a complex network of miRNAs governs the expression of genes associated with heavy metal response [131,132]. For instance, He et al. [133] utilized high-throughput sequencing to identify and compare root miRNA profiles in two *Nicotiana tabacum* cultivars, namely Guiyan 1 (Cd-sensitive) and Yunyan 2 (Cd-resistant). Using quantitative RT-PCR and the miRBase database, they validated known miRNAs and predicted novel ones using bioinformatics tools like RNAfold and Mireap. This genome-wide analysis provided insights into differential miRNA expression under Cd stress and helped identify potential targets involved in Cd tolerance mechanisms.

He et al. [133] employed differential gene expression (DEG) analysis to screen candidate genes in tobacco and subsequently used the psRNA target tool to predict potential miRNA targets. They revealed that Cd stress significantly altered miRNA (and miRNA length) profiles across both cultivars, suggesting broad regulatory roles of miRNAs under Cd exposure. They identified 72 known and 14 novel differentially expressed miRNAs, of which 28 known and 5 novel miRNAs were associated with Cd tolerance. Interestingly, the Cd-tolerant cultivar (Yunyan 2) accumulated higher levels of Cd in roots and shoots compared to the sensitive cultivar (Guiyan 1), yet it maintained superior growth and physiological performance. This indicates that tolerance is not solely determined by metal uptake but also by efficient detoxification, sequestration, and activation of stress-response mechanisms that mitigate Cd toxicity.

Interestingly, the Cd-tolerant cultivar Yunyan 2 accumulated more Cd than the Cd-sensitive Guiyan 1, yet it showed superior tolerance. This was evident by its lesser reduction in plant height (16.19%) and SPAD value (a chlorophyll indicator, 54.44%) compared to Guiyan 1, which showed reductions of 27.75% and 60.22%, respectively. Based on physiological and molecular assessments, He et al. [133] assigned integrated stress scores of 43.173 for Guiyan 1 and 35.077 for Yunyan 2, confirming Yunyan 2’s higher tolerance (Table 2).

The study also expanded the known tobacco microRNome by identifying novel miRNAs potentially involved in Cd stress responses. The Cd tolerance observed in Yunyan 2 was linked to its distinct miRNA expression profile, suppressing specific miRNAs, facilitating better ROS scavenging and reducing toxicity. This regulatory network further impacted processes such as cell growth, ion balance, redox regulation, hormone signaling, and stress defense, contributing to the enhanced resilience of Yunyan 2 over Guiyan 1 (Table 2).

One miRNA, miR390, plays a critical role in Cd stress responses and is conserved across species like *Oryza sativa*, *Arabidopsis thaliana*, and *Zea mays* [129]. In *O. sativa*, miR390 targets and regulates a stress responsive leucine-rich repeat receptor like kinase gene (*OsSRK*) via mRNA cleavage. Ding et al. [129] investigated this relationship using transgenic *O. sativa* plants overexpressing miR390 (Table 2). These plants exhibited significantly elevated miR390 levels and a concurrent decrease in *OsSRK* transcript levels, validating successful genetic modification.

In a study by Qiu et al. [134], Real-Time PCR was employed to determine the expression profiles of microRNAs in wheat under Cd stress. Using the psRNATarget webtool, the researchers identified potential mRNA targets of these miRNAs. Their findings revealed distinct expression patterns of miRNAs between the roots and shoots of Cd-treated seedlings. Physiological analyses showed significant stress symptoms, including reduced root and shoot elongation, lower leaf relative water content (67% in treated vs. 94–96% in controls after 48 h of Cd exposure), decreased chlorophyll levels, and increased membrane lipid peroxidation—indicators of oxidative stress (Table 2). Among the differentially expressed miRNAs, miR398 was of particular interest. It was found to target CSD (Cu/Zn Superoxide Dismutase), an enzyme involved in ROS scavenging. Under Cd stress, miR398 expression decreased, which in turn elevated CSD levels [134]. In wheat, Cd-induced downregulation of miR398 elevated CSD activity. This increase, while enhancing superoxide (O_2_^−^) detoxification, simultaneously produced higher levels of hydrogen peroxide (H_2_O_2_). Such patterns have been experimentally confirmed in several plant species, where Cd exposure elevated both SOD activity and H_2_O_2_ accumulation, often accompanied by enhanced lipid peroxidation [134,135,136]. These findings suggest that while CSD upregulation mitigates O_2_^−^ toxicity, it may inadvertently exacerbate oxidative stress unless balanced by efficient peroxidase or CATs activity. This suggests that miR398 plays a central role in ROS detoxification by regulating CSD expression and balancing oxidative homeostasis in wheat under Cd exposure. Qiu et al. [134] concluded that miRNAs in wheat are integral components of the Cd stress response signaling network. The organ specific expression of miRNAs between roots and leaves implies a spatial regulation mechanism, where miRNAs perform distinct physiological roles depending on the plant tissue involved in Cd detoxification and tolerance.

In a complementary approach, Zhou et al. [137] explored the regulatory mechanisms between microRNAs HMAs in *T. aestivum* to better understand Cd stress responses. Two contrasting wheat cultivars were analysed: L17, a low-Cd accumulator, and H17, a high-Cd accumulator. Through transcriptomic and miRNA sequencing, the study characterized the miRNA expression profiles of both cultivars under Cd exposure. qRT-PCR was subsequently used to validate and assess the expression of selected miRNAs and *TaHMA* genes in the root tissues. The study revealed distinct differences in miRNA expression between the cultivars. Two microRNAs, namely miR9664-3p and tea-miR159a, were found to be upregulated in L17 under Cd stress (L17Cd) compared to its control (L17CK) but downregulated in H17Cd plants. This inverse expression pattern suggests a cultivar-specific miRNA-mediated regulatory response to Cd. Further analysis indicated that the putative target genes of these two miRNAs were downregulated in L17Cd, reinforcing their regulatory role [137].

Previous research by Shiv et al. [138] showed that tea-miR159a regulates *TaMYB3* (*Myeloblastosis*) expression, playing a positive role in defence against *Puccinia striiformis*, a fungal wheat pathogen, suggesting its involvement in broader stress response pathways. Zhou et al. [137] also identified 32 *TaHMA* genes in *T. aestivum*, which are involved in metal transport and detoxification. Their findings proposed that miRNAs may regulate *TaHMA* expression, particularly *TaHMA2.1*, a gene implicated in Cd transport and tolerance, although further functional validation is still needed.

Together, the studies by Qiu et al. [134] and Zhou et al. [137] highlight miRNAs as crucial regulators of Cd stress responses in wheat (Table 2). Whether by modulating oxidative stress through ROS scavenger pathways or by regulating metal transporter genes such as *TaHMA2.1*, miRNAs represent a key epigenetic layer in enhancing plant resilience against Cd toxicity.

**Table 2:** miRNA-mediated responses to cadmium stress in different plant species.

Species	Findings	Citation
*Nicotiana tabacum* (Guiyan 1 vs. Yunyan 2)	High-throughput sequencing identified 72 known and 14 novel miRNAs differentially expressed. Twenty-eight known and five novel miRNAs were linked to tolerance. Cd accumulation was higher in Yunyan 2, but this cultivar showed smaller reductions in height and chlorophyll content, and a lower integrated stress score, suggesting cultivar-specific miRNA regulation.	[133]
*Oryza sativa*	Transgenic overexpression of miR390 elevated miR390 levels and reduced its target OsSRK transcript, confirming its role in cleaving a stress-responsive receptor kinase.	[129]
*Triticum aestivum*	qRT-PCR and psRNATarget analysis revealed organ-specific miRNA changes. Downregulation of miR398 increased CSD (Cu/Zn-SOD) but also H_2_O_2_, highlighting miR398’s role in balancing ROS detoxification.	[134]
*Triticum aestivum* (L17 vs. H17)	miRNA-seq and transcriptomics in low-Cd (L17) and high-Cd (H17) cultivars identified inversely regulated miR9664-3p and tea-miR159a. Thirty-two TaHMA genes were identified, with miRNA-mediated regulation of HMAs (e.g., TaHMA2;1) implicated in Cd sequestration and tolerance.	[137]

## 11. Long Non-Coding RNAs (lncRNAs) in Heavy Metal Stress Response

Long non-coding RNAs (lncRNAs) directly participate in epigenetic regulation by guiding chromatin modifying complexes to specific genomic loci. LncRNAs are increasingly recognized as pivotal players in regulating plant responses to heavy metal stress. Unlike protein coding genes, lncRNAs, typically longer than 200 nucleotides, are not translated into proteins but play essential roles in regulating gene expression at transcriptional and post-transcriptional levels [139]. The regulatory functions of lncRNAs are particularly crucial in the context of abiotic stressors, including heavy metals such as Cd, Pb, and As.

Research indicates that lncRNAs can modulate stress responses by orchestrating complex regulatory networks involving protein-coding genes, miRNAs, and epigenetic modifications [140]. For instance, lncRNAs can interact with chromatin-modifying complexes and transcription factors, thereby influencing the expression of target genes under heavy metal stress. One prominent lncRNA associated with heavy metal responses is found in *Medicago truncatula*, which plays a role in the plant’s adaptation mechanisms through regulatory networks involving metal tolerance genes [141].

The expression patterns of lncRNAs in response to heavy metal stress highlight their potential role in facilitating plant adaptation and resilience. A study in *Chenopodium quinoa* demonstrated specific lncRNAs that exhibited altered expression in response to salinity stress, showcasing their involvement in stress adaptation [142]. Similarly, in cotton (*Gossypium hirsutum*), lncRNAs were found to exhibit changes in expression in response to drought stress, suggesting a broader role for these molecules in various stress contexts, including heavy metal exposure [140].

Moreover, the interplay between lncRNAs and miRNAs adds another layer of complexity in understanding plant stress responses. LncRNAs can regulate miRNAs through several mechanisms. One of the most studied roles is that lncRNAs act as “molecular sponges” or competing endogenous RNAs (ceRNAs): they contain sequences complementary to specific miRNAs and bind them, thereby preventing the miRNAs from attaching to their actual mRNA targets. In this way, lncRNAs can indirectly increase the expression of genes that would otherwise be silenced by those miRNAs. Additionally, some lncRNAs can influence miRNA biogenesis, either promoting or inhibiting the processing of primary miRNAs into their mature forms. Through these mechanisms, lncRNAs add an extra layer of regulation to gene expression networks, modulating plant responses to stress, development, and other physiological processes [143]. The interactions between these non-coding RNA types facilitate the fine-tuning of gene expression during stress conditions, reflecting a sophisticated regulatory strategy that plants employ to manage heavy metal toxicity [144].

Heavy metal exposure triggers various physiological changes in plants, leading to oxidative stress and genotoxic damage. In response, lncRNAs can be activated as part of the stress response, coordinating the expression of genes involved in detoxification processes and antioxidative mechanisms [145,146]. Studies have demonstrated that lncRNAs are involved in regulating the expression of metal tolerance proteins and polyamines, which play roles in mitigating oxidative stress [147].

Recent advancements in high-throughput sequencing technologies have enabled comprehensive analyses of lncRNA expression profiles under heavy metal stress. These studies suggest that the expression of specific lncRNAs correlates with critical stress associated pathways, such as those governing antioxidant defenses and metal ion transport [146]. The identification of lncRNAs that interact with stress-responsive TFs offers promising avenues for genetic engineering aimed at enhancing heavy metal tolerance in crops [148].

To further understand the functional roles of lncRNAs in heavy metal stress responses, it is essential to employ integrative approaches that combine transcriptomic, proteomic, and metabolomic analyses. Such studies will help elucidate the intricate regulatory networks involving lncRNAs, providing insights into their molecular mechanisms of action [149,150]. This comprehensive understanding can inform breeding programs aimed at developing heavy metal-tolerant crop varieties through the manipulation of lncRNA expression. Several manuscripts are already available that describe the role of lncRNAs in heavy metal stress responses (Table 3).

In rice (*Oryza sativa* Indica “Huanghuazhan”), hydroponic exposure to Cd and As alters the expression of over 3300 lncRNAs, with specific transcripts, such as MSTRG.24054.4, showing coordinated, cis-acting regulation of nearby detoxification genes under Cd/As stress [151].

Similarly, in Tibetan wild barley (*Hordeum vulgare*), aluminium (Al) treatment triggers differential expression of 268 lncRNAs, which form cis-acting lncRNA:mRNA pairs enriched in pathways like peroxisome function and diterpenoid biosynthesis. These lncRNAs are implicated in transcriptional and chromatin-level modulation of Al tolerance genes [152].

In *Betula platyphylla*, Wen et al. [153] conducted a genome-wide survey of lncRNAs under Cd stress and identified 30 transcripts whose expression was significantly altered compared to control conditions. Remarkably, two of these lncRNAs enhanced Cd tolerance, whereas two others increased sensitivity, underscoring a nuanced regulatory landscape in which individual lncRNAs can play opposing roles in stress adaptation. Downstream target analysis revealed four protein-coding genes, namely *l-Lactate Dehydrogenase A* (*LDHA*), *Heat Shock Protein* (*HSP18.1*), *Yellow Stripe-like protein* (*YSL9*) and *H/ACA Ribonucleoprotein Complex Subunit 2-like Protein* (*HRCS2L*), that recapitulated similar phenotypic effects upon overexpression, implicating them as likely mediators of lncRNA-dependent Cd response pathways. Together, these findings delineate a set of Cd-responsive lncRNAs in a woody species and link them to specific detoxification and homeostasis mechanisms, providing a foundation for further mechanistic dissection.

Similarly, Gui et al. [141] catalogued 3.284 lncRNA loci in *Medicago truncatula* and found 515 to be Al-responsive upon genome-wide profiling. Two candidates, *ALMT-related DElncRNAs* (MSTRG.12506.5 and MSTRG.34338.20), when overexpressed in yeast, each conferred enhanced Al tolerance, demonstrating functional roles in resistance in a heterologous system. Many Al-responsive lncRNAs map in *cis* near *Malic Acid Transporter* (*ALMT*) genes, and pathway analysis highlighted hormone signaling, cell-wall modification, and the Tricarboxylic Acid cycle (TCA) as enriched processes. Although these findings support a *cis*-regulatory model for lncRNA-mediated Al adaptation, the authors did not investigate chromatin remodelling or RNA-directed DNA methylation mechanisms.

Moreover, in *Hordeum vulgare* roots of the Cd-tolerant genotype ZN8 and the sensitive W6nk2, Zhou et al. [154] identified 9937 novel lncRNAs and predicted 5758 *cis*-acting and 4159 *trans*-acting lncRNA: mRNA interactions upon 5 µM Cd treatment. Under Cd stress, ZN8 displayed 572 up- and 567 down-regulated lncRNAs, whereas W6nk2 genotype showed 178 up- and 214 down-regulated transcripts, with 195 lncRNAs differing between the two genotypes. Integration of miRNA target predictions revealed 8 lncRNAs functioning as miRNA mimics, including those targeting the R2R3-MYB factor HvGAMYB, whose silencing via barley stripe mosaic virus-induced gene silencing led to Cd hypersensitivity, increased metal accumulation, and disrupted photosynthetic and antioxidant gene expression. These results delineate a multilayered lncRNA: mRNA network underpinning Cd tolerance in barley.

In Tibetan hull-less barley roots of the Cd-tolerant genotype X178 and the sensitive X38, RNA-Seq uncovered 8299 novel lncRNAs linked to 5166 target genes, with 1884 regulated in *cis* and 3428 in *trans*. Differential expression analysis under Cd stress pinpointed 26 lncRNAs and 150 mRNAs likely tied to tolerance, and functional enrichment highlighted detoxification and stress-response pathways particularly aromatic amino acid metabolism, ABC transporters and secondary metabolite biosynthesis. Of these, 12 lncRNAs formed 18 lncRNA: mRNA regulatory pairs predicted to modulate proteins such as DJ-1 homolog B (DJ-1), Protein-Enhanced Disease Resistance (EDR), Putrescine Hydroxycinnamoyl Transferase 1 (PHT), and ABC transporters (ABC-T). Among these, ABC transporters and PHT are particularly relevant for heavy metal tolerance, as they mediate Cd sequestration and detoxification processes. Likewise, DJ-1, a redox-sensitive protein, is implicated in mitigating oxidative stress, while EDR proteins contribute to maintaining cellular homeostasis under stress. qRT-PCR validation confirmed the reliability of these candidate interactions, suggesting that coordinated regulation of detoxification (ABC-T, PHT), oxidative stress defense (DJ-1), and stress signaling pathways (EDR) underpins the superior Cd tolerance observed in the barley cultivar X178 [155].

In *Cajanus cajan* (pigeon pea), two complementary modules were shown to enhance Al tolerance via citrate metabolism. First, the transcription factor CcNFYB3 (Nuclear transcription factor Y subunit beta-3) binds the CcMATE35 (Multidrug and toxic compound extrusion 35) promoter to boost citrate efflux under Al stress, resulting in improved root elongation and reduced cell death in CcNFYB3-OE and CcMATE35-OE lines. Second, the lncRNA CcLTCS (Long noncoding RNA Targeting Citrate Synthase) upregulates *CcCS* (*Citrate Synthase*), elevating root citrate synthesis and conferring greater Al resistance in CcLTCS-OE plants. Co-overexpression of both modules yields a synergistic increase in citrate efflux and synthesis, highlighting their joint role in pigeon pea Al detoxification [156].

Moreover, in *Oryza sativa* L. (“Huanghuazhan”) seedlings grown hydroponically with 10 µM CdCl_2_, supplementation with 250 mg L^−1^ melatonin reduced shoot Cd accumulation by 30%, improved K^+^ and Ca^2+^ uptake by 21.2%, increased net photosynthetic rate by 164.5%, and lowered malondialdehyde (MDA) levels by 33.2%. Transcriptome profiling revealed 2510 differentially expressed transcripts under Cd+MLT treatment, including six key lncRNAs (e.g., LOC107279206, BGIG39947_32145) that interact with mRNAs encoding cell-wall–modifying enzymes and photosynthetic proteins; these networks underpin melatonin’s enhancement of Cd tolerance via modulation of cell-wall composition, photosynthesis, and redox homeostasis [157].

In wheat, Cd stress drives the expression of over 10,000 novel lncRNAs, with 69 predicted to cis-regulate genes involved in Cd transport/detoxification, photosynthesis, and antioxidant defense, changes that mirror altered Cd accumulation, photosystem performance, and ROS levels. Overexpressing lncRNA37228, which targets the photosystem II (PSII) D1 gene, enhances Cd tolerance in *Arabidopsis*, and a genome-wide survey highlights the broader involvement of the PSII protein family in wheat’s Cd response. These results reveal a key role for lncRNA-mediated regulation in adapting to Cd toxicity and pinpoint targets for improving crop safety [158].

Zhao et al. [159] discovered that TalncRNA18313 is a Cd-inducible lncRNA in wheat whose expression is strongly upregulated in leaves under Cd stress. Heterologous expression of TalncRNA18313 in *Arabidopsis* enhances Cd tolerance, transgenic lines show reduced lipid peroxidation (lower MDA) and elevated activities of antioxidant enzymes (CAT, SOD, peroxidase). RNA-seq of these overexpressors under Cd stress revealed 370 differentially expressed genes, with significant enrichment in transcriptional regulators and antioxidative defense pathways, indicating that TalncRNA18313 orchestrates gene-expression networks and antioxidant responses to mitigate oxidative damage.

Lin et al. [160] discovered that in sweet sorghum roots, a high-Cd accumulator (‘H18’) versus a low-Cd accumulator (‘L69’), 1988 lncRNAs are expressed, of which 52 and 69, respectively, are differentially regulated by Cd stress. Predictive analyses identified 1888 putative *cis*-target genes and 65 lncRNAs as endogenous target mimics for 117 miRNAs. Dual-luciferase reporter assays (DLR) showed that lncRNA 15962 sequesters sbi-miR5565e, thereby derepressing two cell-wall–metabolism genes, while overexpression of four lncRNAs, including one upstream of the Cd-transporter gene belonging to the yellow stripe-like (YSL) family SbYS1, upregulated their *cis*-targets in protoplasts. Conversely, miRNA-mediated inhibition of lncRNA 11558 in ‘H18’ seedlings decreased SbYS1 expression. These results highlight lncRNAs as positive Cd uptake and translocation regulators in sweet sorghum.

Finally, in Tibetan wild barley, hydroponic exposure to 50 µM Al revealed 268 Al-responsive lncRNAs and 938 predicted cis-acting lncRNA–mRNA pairs. Their target genes are enriched in diterpenoid biosynthesis, peroxisome function, and starch/sucrose metabolism, with 15 lncRNAs uniquely regulated in the Al-tolerant genotype XZ16, highlighting their potential roles in Al tolerance [152].

Together, these studies highlight how heavy metals (e.g., Pb, Cd) and metalloids (elements with both metal- and non-metal-like properties, such as As) trigger lncRNA-mediated epigenetic networks. These networks act through cis-regulation, chromatin remodeling, and interactions with DNA methylation and histone modifiers to fine-tune stress-responsive gene expression in plants.

**Table 3:** lncRNA-Mediated Heavy Metal Stress Responses Across Plant Species.

Species	Findings	Citation
*Oryza sativa* Indica “Huanghuazhan”	Hydroponic Cd and As exposure alters > 3300 lncRNAs; e.g., MSTRG.24054.4 acts in cis on nearby detoxification genes under combined stress. 10 µM CdCl_2_ + 250 mg/L melatonin reduces shoot Cd by 30%, improves K^+^/Ca^2+^ uptake, boosts photosynthesis, lowers MDA; transcriptome shows 2510 DE transcripts including six lncRNAs interacting with mRNAs for cell-wall enzymes and photosynthetic proteins, underpinning melatonin-mediated Cd tolerance.	[151]
*Hordeum vulgare* (Tibetan wild barley)	Al treatment induces 268 lncRNAs forming cis-acting lncRNA–mRNA pairs enriched in peroxisome and diterpenoid-biosynthesis pathways, implicating transcriptional/chromatin-level regulation of Al tolerance genes.	[152]
*Hordeum vulgare* (ZN8 vs. W6nk2)	Identified 9937 novel lncRNAs under 5 µM Cd; 5758 cis- and 4159 trans-acting pairs; eight lncRNAs act as miRNA mimics (e.g., targeting HvGAMYB), with virus-induced silencing of HvGAMYB causing Cd hypersensitivity and disrupted photosynthetic/antioxidant gene expression.	[154]
*Hordeum vulgare* (hull-less, X178 vs. X38)	RNA-Seq revealed 8299 lncRNAs (1884 cis, 3428 trans) under Cd stress; 26 lncRNAs and 150 mRNAs linked to tolerance; 12 lncRNAs form 18 lncRNA–mRNA pairs modulating DJ-1, EDR, PHT, ABC transporters; qRT-PCR validated candidates in genotype X178.	[155]
*Cajanus cajan* (pigeon pea)	Two modules enhance Al tolerance: TF CcNFYB3 → CcMATE35 increases citrate efflux; lncRNA CcLTCS → CcCS boosts citrate synthesis; co-overexpression synergistically improves Al detoxification and root health.	[156]
*Triticum aestivum* (wheat)	Cd stress induces >10,000 novel lncRNAs; 69 cis-regulate genes for Cd transport, photosynthesis, antioxidant defense; overexpression of lncRNA37228 (targets PSII D1) in Arabidopsis enhances Cd tolerance, linking PSII family to wheat Cd response.	[158]
*Triticum aestivum* (wheat)	TalncRNA18313 is Cd-inducible in leaves; heterologous expression in Arabidopsis lowers MDA and raises CAT, SOD, peroxidase activities; RNA-seq of overexpressors under Cd stress identifies 370 DE genes enriched in transcriptional regulation and antioxidative defense pathways.	[159]
*Sorghum bicolor* (sweet sorghum, H18 vs. L69)	lncRNA-seq in roots identified 1988 lncRNAs; 52 and 69 DE in H18 and L69 under Cd; 65 lncRNAs target 117 miRNAs, 1888 cis-genes; lncRNA 15962 sequesters sbi-miR5565e to derepress cell-wall genes; overexpression of four lncRNAs upregulates their cis-targets (including SbYS1); miRNA inhibition of lncRNA 11558 decreases SbYS1, confirming positive regulation.	[160]

## 12. Future Perspectives: Epigenome Editing and Crop Improvement for Heavy Metals Resistance

**Figure 3:**
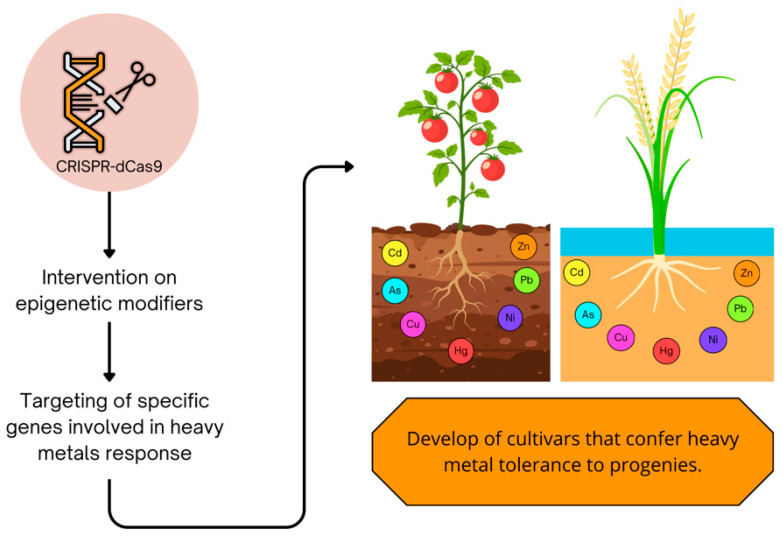
CRISPR-dCas9-based epigenome editing for cadmium stress tolerance in plants.

The potential for epigenome editing to enhance crop improvement for heavy metals resistance holds significant promise in the quest for sustainable agricultural practices (Figure 3). Heavy metal contamination poses a critical threat to food security and ecosystem health, and employing modern biotechnological approaches, particularly epigenetic modifications, can facilitate the development of crops with superior tolerance to metal stress [161,162]. Recent advancements in genomic editing technologies such as CRISPR/Cas have opened new trajectories for precisely manipulating epigenetic marks (Figure 3), thus allowing for targeted improvements in crop varieties [163].

Various studies have highlighted the relevance of epigenetic regulation in crop response to environmental stresses such as heavy metal toxicity. Epigenetic processes, including DNA methylation and histone modifications, profoundly impact gene expression without altering the underlying DNA sequence [161,162]. Harnessing these modifications through targeted epigenome editing presents an advantageous strategy for enhancing plant resilience.

Incorporating epigenetic information into breeding programs can complement existing genetic approaches, which may lead to improved response to heavy metal stress. A study highlighted the potential of epitranscriptomics to provide additional layers of regulatory control that could be exploited to enhance metal tolerance [82]. Such approaches can involve creating hybrid lines that exhibit enhanced tolerance due to combined epigenetic traits derived from parental sources. By elucidating specific lncRNAs and miRNAs involved in metal stress responses, scientists can devise strategies for developing crops that are better equipped to cope with toxic metal levels in soil and water [164].

Moreover, exploring natural epigenomic variations present in crop wild relatives could significantly enhance metal tolerance breeding efforts. Crop wild relatives are considered a valuable genetic resource for improving agronomic traits, as their epigenetic diversity may provide additional resilience mechanisms against heavy metal toxicity [165,166]. Integrating this genetic variation into advanced breeding techniques can facilitate the development of crops capable of thriving in heavy metal-polluted environments while maintaining agricultural productivity.

The application of bioinformatics and statistical genomics is noteworthy in deciphering epigenetic landscapes across different genotypes exposed to heavy metals. With high-throughput sequencing technologies, researchers can conduct detailed mapping of epigenetic modifications across the genome, leading to insights that can guide breeding programs towards selecting Epigenetically favorable traits that enhance plant tolerance to heavy metal stress [167] (Figure 3). Furthermore, the understanding of transgenerational epigenetic inheritance highlights the possibility of developing cultivars that can confer stress tolerance to their progenies, thereby establishing a framework for sustainable crop production and management in contaminated soils [168].

As we pivot towards climate-smart agriculture, leveraging epigenetic knowledge in conjunction with traditional breeding and genetic engineering will be essential. Specific epigenetic markers, heritable chemical modifications to DNA or histone proteins that regulate gene activity without changing the underlying DNA sequence, can be unraveled, facilitating more efficient breeding strategies for heavy metal resistance [169]. Emphasizing multidisciplinary collaboration will be crucial in addressing the complex challenges posed by heavy metals in agriculture. Environmental factors, soil amendments, and microbial interactions all play significant roles in the efficacy of crop resilience strategies [170,171].

Looking ahead, more focused research efforts are required to couple epigenome editing with agronomic practices that minimize heavy metal uptake by crops. Strategies such as optimizing soil health, implementing efficient water management practices, and utilizing plant-associated microbes (e.g., plant growth-promoting rhizobacteria) as facilitators of stress tolerance can synergistically enhance the effectiveness of epigenetic approaches [171,172,173]. By fostering a comprehensive view of plant responses and integrating these practices, significant strides can be made towards achieving sustainable agricultural systems capable of thriving amid heavy metal contamination.

## 13. Conclusions

In summary, plants confronted with heavy-metal stress deploy a multilayered epigenetic toolkit, encompassing DNA methylation dynamics, histone modifications, chromatin remodelling, and non-coding RNA networks (miRNAs and lncRNAs), to reprogram stress-responsive genes and safeguard cellular homeostasis. These modifications not only mediate immediate detoxification and antioxidant defences but can also be stably inherited, endowing progeny with a “memory” of ancestral exposure that enhances tolerance [104,174,175]. Emerging evidence for Cd-induced reprogramming of miRNAs and lncRNAs further reveals how RNA-mediated pathways fine-tune metal-transporter expression, ROS scavenging, and cell-wall metabolism across generations [176,177]. Together, these insights underscore the promise of targeting epigenetic marks, via selective breeding or CRISPR-based epigenome editing, to create crop varieties with durable heavy-metal resilience, offering a sustainable strategy to safeguard agricultural productivity and ecosystem health in contaminated environments.

## Figures and Tables

**Table 1:** Natural and anthropogenic sources of heavy metal.

Metal	Sources	Citations
Cadmium	Sedimentary rocks and marine phosphates, production of alloys, pigments, batteries, mining operations, industrial manufacturing, phosphate-based fertilizers	[60,66]
Mercury	Coal combustion, industrial processes (production of caustic soda, nuclear reactors, antifungal agents, solvent for reactive and precious metal) electrical industry (switches, thermostats, batteries), cement production	[60,67]
Arsenicum	Volcanic eruptions and soil erosion, mining runoff, agricultural pesticides, dyestuffs	[60,68]
Lead	Industrial emissions, lead-based paints, batteries, fossil fuels burning, mining, manufacturing	[60,69]
Copper	Industrial discharges, farming activities, corrosion of plumbing systems, alloys, electric circuit boards, electromagnets	[60,70]
Zinc	Industrial waste, mining by-products, sewage sludge, Ni-Zn batteries, manufacture of plastics, paints	[60,70]

## Data Availability

In this work, no new data were created.

## Abbreviations

Pytochelatins (PCs); metallothioneins (MTs); superoxide dismutase (SOD); catalase (CAT); reactive oxygen species (ROS); clustered regularly interspaced short palindromic repeats (CRISPR); Programmed Cell Death (PCD); Small RNAs (sRNA); microRNAs (miRNAs); Non-coding RNAs (ncRNAs); RNA-directed DNA methylation (RdDM); DNA methyltransferases (DNMTs); histone acetyltransferases (HATs); histone deacetylases (HDACs); long non-coding RNAs (lncRNAs); plant small RNA (psRNA); malondialdehyde (MDA); Dual-luciferase reporter assays (DLR).
